# Unsupervised Denoising in Spectral CT: Multi-Dimensional U-Net for Energy Channel Regularisation

**DOI:** 10.3390/s24206654

**Published:** 2024-10-16

**Authors:** Raziye Kubra Kumrular, Thomas Blumensath

**Affiliations:** Institute of Sound and Vibration Research, Department of Engineering and the Environment, University of Southampton, University Rd., Southampton SO17 1BJ, UK

**Keywords:** deep learning, spectral computed tomography, unsupervised denoising method

## Abstract

Spectral Computed Tomography (CT) is a versatile imaging technique widely utilized in industry, medicine, and scientific research. This technique allows us to observe the energy-dependent X-ray attenuation throughout an object by using Photon Counting Detector (PCD) technology. However, a major drawback of spectral CT is the increase in noise due to a lower achievable photon count when using more energy channels. This challenge often complicates quantitative material identification, which is a major application of the technology. In this study, we investigate the Noise2Inverse image denoising approach for noise removal in spectral computed tomography. Our unsupervised deep learning-based model uses a multi-dimensional U-Net paired with a block-based training approach modified for additional energy-channel regularization. We conducted experiments using two simulated spectral CT phantoms, each with a unique shape and material composition, and a real scan of a biological sample containing a characteristic K-edge. Measuring the peak signal-to-noise ratio (PSNR) and structural similarity index (SSIM) for the simulated data and the contrast-to-noise ratio (CNR) for the real-world data, our approach not only outperforms previously used methods—namely the unsupervised Low2High method and the total variation-constrained iterative reconstruction method—but also does not require complex parameter tuning.

## 1. Introduction

Spectral Computed Tomography (CT) imaging has been a very active field of research, as it allows us to observe the energy dependence of the object being imaged using Photon Counting Detector (PCD) technology [1]. The applicability of PCD technology to obtain energy-resolved images has been shown in different fields. The first clinical PCD-CT system has demonstrated better resolution and noise characteristics in four different clinical applications than similarly configured energy-integrating CT (EID-CT) [2]. Spectral imaging is also widely used in threat detection during airport luggage security screening [3,4], as well as in different applications of Non-Destructive Testing (NDT) [5,6].

In spectral imaging, the projection data are intrinsically noisy because there are fewer photons in each energy channel. The energy channel must be carefully chosen to minimise noise because wider energy channels integrate more photons and, thus, have a lower noise level. Consequently, there is a trade-off between the width of energy channels and the noise level. To address these challenges, specialized noise-robust spectral CT reconstruction techniques have been developed. Some of these methods focus on total variation (TV)-based methods [7], dictionary learning methods [8], prior-based methods [9], and tensor-based nuclear norm regularization [10]. However, these studies [8,9,10] used detectors with a maximum of eight energy channels, each with a width of several keVs, which is far from the ideal assumption of monochromatic acquisition. To manage the larger amounts of data from more energy channels, 2D reconstruction with TV was applied in [7], but the required regularization tuning makes this approach time-consuming and less practical. Although more recent detectors provide a significantly finer energy resolution, increasing the number of energy channels leads to significant computational challenges when using the above iterative algorithms, which operate jointly across the channels.

Thus, investigating alternative approaches based on channel-wise reconstruction remains crucial, especially when working with large multi-channel datasets. Recently, data-driven approaches have been applied for spectral imaging. A supervised deep learning-based spectral CT method that includes information in the spectral domain was designed to improve reconstructions when the signal is affected by Poisson noise [11]. The challenges of obtaining high-quality reconstructions from sparse measurements for a 64-channel PCD-CT were addressed using an unsupervised denoising method called Low2High [12] that can be applied after independent, single-channel reconstructions. In this study, we leverage the Noise2Inverse [13] framework in combination with a multi-dimensional U-Net architecture [14], employing a block-based training approach [15]. This method enables training on pairs of noisy images, each reconstructed from mutually exclusive subsets of projections, thereby eliminating the need for clean training data. Given the significant challenges in acquiring clean and diverse datasets for spectral imaging, particularly due to the inherent noise in spectral CT data, self-supervised approaches like ours offer a robust alternative. Our approach aims to improve image quality and, thus, help in accurate material identification in spectral imaging applications where clean training datasets are not available.

## 2. Background

### 2.1. Spectral Imaging

The attenuation of an X-ray beam travelling through an object is often modelled using the Beer–Lambert law. For a poly-energetic X-ray spectrum used in spectral Imaging, an adapted version of the Beer–Lambert law is expressed as follows:

(1) $$I\left(E\right)=\;{I_{0}\left(E\right)e}^{-\int_{L}\mu \left(E,r\right)dr},$$

where I(E) and $I_{0}\left(E\right)$ are the transmitted (measured) X-ray intensity and the initial intensity emitting from the X-ray source, respectively, at an energy level of *E* respectively, both of which include the detector sensitivity. $\mu \left(E,r\right)$ is the linear attenuation coefficient (LAC) of the object at an energy level of *E*, and $\int_{L}\mu \left(E,r\right)dr$ represents the line integral of attenuation along one ray path from the source to one detector element at one rotation angle. This line integral sums attenuation along the path (*r*). It is critical to note that both the energy dependency of the X-ray source spectrum ($I_{0}\left(E\right)$) and the energy sensitivity of the detector significantly influence the system’s overall spectral response. Consequently, Spectral CT images are generated by the individual tomographic reconstruction of each energy channel using the measured sinograms (I(E), i.e., projections), which enables the incorporation of detailed energy information into the images [1,6].

### 2.2. Unsupervised Learning Methods

Data-driven approaches to image denoising can be divided into the following three categories: unsupervised, supervised, and semi-supervised methods. Here, we focus on unsupervised methods, since there is often a lack of low-noise, high-quality reference data in CT imaging applications [13,16] that could be used for supervised training. Spectral CT applications exemplify this challenge. Thus, denoising methods that may be trained with noisy reference data of paired images [17] or a single image [18] become of interest. There are a few strategies for this. In the Noise2Noise training method [17], a pair of images is used, both showing the same object but where each image contains independent, zero mean noise. Such pairs are generally unavailable in CT. This drawback is eliminated by the Noise2Self method [18], which uses a single noisy image. However, it utilises the assumption that noise in one pixel is statistically independent of noise in another pixel, which is not the case in tomographic reconstructions. The Noise2Inverse approach does not require spatially independent noise or matched image pairs [13,16]. Instead, it utilises the realisation that most noise in tomographic measurements is independent from projection to projection. By splitting the projection data into two mutually exclusive sets, two images can be reconstructed with independent noise. A model is then trained to predict one noisy image from the other noisy image. Under the assumption that the image structure is predictable but the noise is not, the model then learns to predict a noise-free image.

Describing this approach in detail, we use the Beer–Lambert law to convert the X-ray intensity measurements to absorption values and discretise the integral as follows:

(2) $$\tilde{y}=Ax+\epsilon ,$$

where $\tilde{y}$ contains the absorption measurements corrupted by noise, that is, element-wise independent and zero-mean conditional on the data. *A* represents the projection operator, and *x* is the discretised absorption image. Noise2Inverse is implemented in single-energy tomographic reconstruction by reconstructing image pairs from mutually exclusive subsets of the measurement data. Reconstructed noise in the image pairs is then assumed to be independent.

For denoising, we use a parameterised deep neural network ($\Lambda_{\theta }$) that is trained by optimising the parameters as follows:

(3) $$\theta^{★}=\arg\min_{\theta }\frac{1}{\left|J\right|}\sum_{J\in J}{∥\Lambda_{\theta }\left({\tilde{x}}_{J^{C}}\right)-\left({\tilde{x}}_{J}\right)∥}_{2}^{2},$$

providing the best prediction of the target reconstruction (${\tilde{x}}_{J}$; reconstructed from the projections in set *J*) from the input (${\tilde{x}}_{J^{C}}$; reconstructed from the projections in set $J^{C}$).

Based on similar reasoning, the Low2High [12] approach was introduced for sparse multi-spectral imaging. In the Low2High method, a different strategy is used to produce two pairs of images with independent noise generated from the same set of measurements. This is done using a filtered backprojection (FBP) [19] algorithm that uses two different filters, namely the standard FBP Ram-Lak (s=1) filter (high) and a Hann (s=0.2) low-pass filter that removes the higher image frequencies (low). The reasoning here is that noise is predominately concentrated at higher frequencies so that the low-frequency image does not contain significant noise and the network is then trained to predict the coherent high frequencies from the low-frequency content whilst the noise averages out in the same way as in Noise2Inverse [13,16].

### 2.3. Our Approach

**Figure 1:**
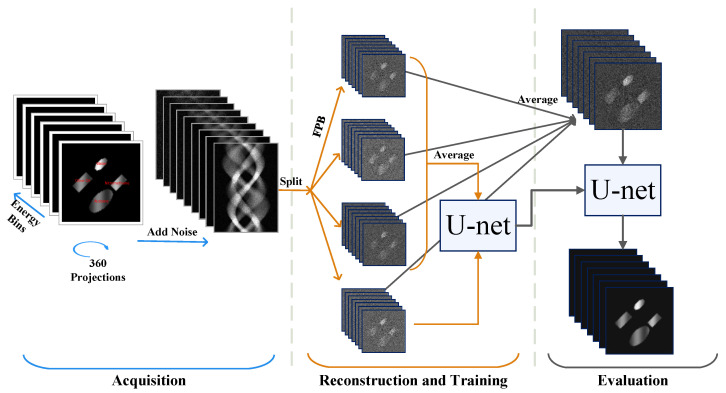
Our approach: The spectral sinograms are obtained over $360^{\circ }$ and split into 4 (K=4) mutually exclusive sets, which are reconstructed independently for each energy channel using FBP. The network is trained using images generated by averaging all possible combinations of 3 reconstructions out of the 4 images as network input to predict the 4th spectral image not used to generate the current network input. Once trained, all 4 images are averaged and denoised by the model. The workflow is divided into three main stages, each distinguished by a unique colour.

The Noise2Inverse approach is well-suited for denoising in tomographic imaging, and our approach proposes an unsupervised learning strategy for spectral imaging based on the Noise2Inverse method. The approach requires us to generate input and target images with independent noise. To achieve this, for a given set of projections acquired over an angular range of $360^{\circ }$, we split the sinogram into *K* different subsets ($({\tilde{y}}_{E_{1}}$, …,${\tilde{y}}_{E_{N}}{)}_{1}$, …,$({\tilde{y}}_{E_{1}}$, …,${\tilde{y}}_{E_{N}}{)}_{K}$), where each split contains mutually exclusive projections at equally spaced angles for the same energy channels. After splitting the sinogram, each subset of the sinogram is reconstructed using energy channel-wise FBP ($({\tilde{x}}_{E_{1}}$, …,${\tilde{x}}_{E_{N}}{)}_{1}$, …,$({\tilde{x}}_{E_{1}}$, …,${\tilde{x}}_{E_{N}}{)}_{K}$). For the training step, we generate *K* different network input images by averaging over all K−1 different subgroups of reconstructions, where each subgroup contains K−1 images, whilst the target image is the reconstruction from the set not included in the network input. With this strategy, the input is less noisy than the target. To estimate the final denoised image ($(x_{E_{1}}^{*}$, …,$x_{E_{N}}^{*}{)}_{1}$, …,$(x_{E_{1}}^{*}$, …,$x_{E_{N}}^{*}{)}_{K}$), all inputs used in training are averaged and used as input for the trained network. A schematic diagram of our approach for K=4 is detailed in Figure 1 based on the methodologies used in the synthetic data experiment. The differences between the real and synthetic data experiments include the dimensions of projections and models, the selected subsets, and the use of the FDK reconstruction algorithm instead of the FBP algorithm.

### 2.4. Evaluation Metrics for Image Quality

This section discusses evaluation metrics that are commonly used in the literature to measure image quality and are also employed in this paper. The comparative analysis of these metrics allows us to distinguish their relative efficacy in methods addressed in this paper.

- The Peak Signal-to-Noise Ratio (PSNR) can be used as a measure of performance. PSNR measures image quality by calculating the ratio between the maximum of the signal power and the power of the noise. The PSNR value is expressed in decibels due to the wide dynamic range. The PSNR between images *f* and *g* is computed as follows:
  (4) $$\mathrm{PSNR}\left(f,g\right)=20\cdot \log_{10}\left(\max\left(f,g\right)\right)-10\cdot \log_{10}\left(\mathrm{MSE}\left(f,g\right)\right),$$
  The Mean Square Error (MSE) between image *f* and image *g* can be calculated as follows:
  (5) $$\mathrm{MSE}\left(f,g\right)=\frac{1}{mn}\sum_{i=1}^{m}\sum_{j=1}^{n}{\left(f\left(i,j\right)-g\left(i,j\right)\right)}^{2},$$
  where *f* is the ground truth and *g* is the reconstructed/denoised image. If the MSE approaches zero, the PSNR value approaches infinity; this suggests that a higher PSNR value provides higher image quality. Furthermore, a small PSNR value indicates high numerical differences between the images [20]. In general, a PSNR value greater than 20 dB is considered to indicate good image quality.
- The structural similarity index (SSIM) is a quality metric used to measure the similarity between two images [21]. This metric is correlated with the perception of the human visual system. The SSIM is defined as follows:
  (6) $$\mathrm{SSIM}\left(f,g\right)=\frac{\left(2\mu_{f}\mu_{g}+c_{1}\right)\left(2\sigma_{fg}+c_{2}\right)}{\left(\mu_{f}^{2}+\mu_{g}^{2}+c_{1}\right)\left(\sigma_{f}^{2}+\sigma_{g}^{2}+c_{2}\right)},$$
  where $\mu_{f}$ and $\mu_{g}$, $\sigma_{f}$ and $\sigma_{g}$, and $\sigma_{fg}$ represent the local means, standard deviations, and cross-covariance of images *f* and *g*, respectively. Regularization constants $c_{1}$ and $c_{2}$ are very small values that prevent zeros in the denominator. The SSIM value varies between 0 and 1. An SSIM index close to 1 shows an ideal agreement between the images, whilst a value of 0 shows no correlation between the images [20,21].
- The contrast-to-noise ratio (CNR) is a widely used metric to evaluate imaging quality. It helps to understand how easily low-contrast objects can be distinguished from their surroundings [22,23]. The CNR is calculated by dividing the signal difference between the target region and the background by the sum of the noise in both regions (Equation (7)) [24]. In particular, the larger the signal difference and the smaller the noise, the higher the CNR, which indicates better image quality [22,23].
  (7) $$CNR=\frac{|S_{T}-S_{B}|}{\sigma_{T}+\sigma_{B}},$$
  where $S_{T}$ and $S_{B}$ represent the average of selected area and background in the reconstructed/denoised image, respectively, while $\sigma_{T}$ and $\sigma_{T}$ stand for the standard deviation (SD) of the selected area and background area, respectively.

## 3. Methodology

### 3.1. Synthetic Spectral CT Data

**Figure 2:**
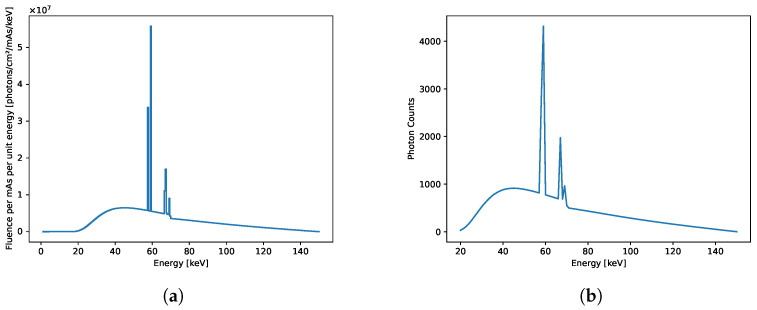
(**a**) Source spectrum obtained with SpekPy at 150 kVp. The source spectrum has a maximum value of $5.58\times 10^{7}\;{\mathrm{photons}/\mathrm{cm}}^{2}/\mathrm{mAs}/\mathrm{keV}$, representing the peak fluence per unit of energy. (**b**) Normalised X-ray source spectrum over the energy range of 20 to 150 keV. The y axis represents the number of photons, while the x axis indicates the energy level in keV for both spectra.

To simulate an X-ray source spectrum, we used SpeckPy software (v2.0) [25]. The simulation used a tube voltage of 150 kVp and a tungsten reflection target at an angle of 12 degrees with filtering of 4 mm aluminium, 1 mm beryllium, and 1000 mm air. The width of the energy bin [keV] was selected as 0.5 keV, and the exposure setting was selected as 1 mAs. To simulate a spectral resolution of 1 keV, we created 131 spectral energy bins between 20 and 150 keV. Specifically, we interpolated the source spectrum between 19.550 and 150.450 keV with an initial resolution of 0.1 keV before averaging the X-ray flux over 10 neighbouring energy bands. To normalise the X-ray fluence of the source, we assumed an X-ray exposure time that would guarantee the detection of 60,000 photons for each pixel when summed over all energy channels. Figure 2a illustrates the source spectrum obtained using SpekPy [25], while Figure 2b demonstrates the normalised X-ray source spectrum with the number of photons per energy bin on the y axis in the energy range of 20 to 150 keV. In both spectra, the y axis shows the number of photons, and the x axis represents the energy levels in keV.

**Figure 3:**
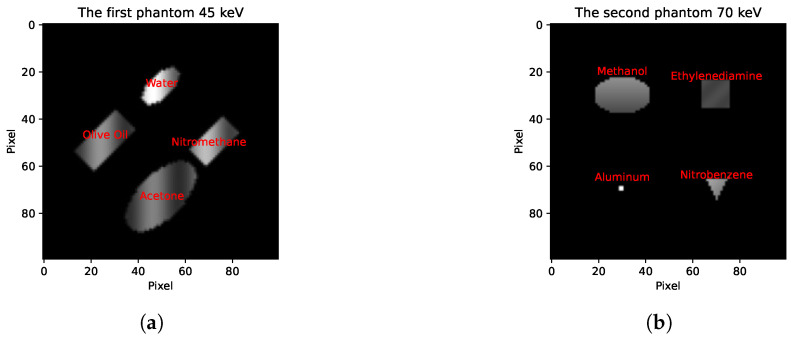
Examples of phantoms at energy levels of 45keV and 70keV, featuring different materials and shapes: (**a**) water, olive oil, nitromethane, and acetone; (**b**) methanol, ethylenediamine, aluminium, and nitrobenzene.

We created two 2D phantoms (each with spatial dimensions of 100 × 100) containing four distinct objects, with each object composed of a different material. We utilised the X-ray DB Python library, which provides attenuation profiles of materials for various elements and compounds, to simulate our phantoms. We sourced the densities of the selected materials from the PubChem database [26]. Densities were spatially modulated using a sinusoidal function to simulate relative density variations throughout the object. We assigned the X-ray attenuation coefficients of the material to objects (shown in Figure 3) and the background to zero. The choice of the 8 materials used in the experiment and the selection of the spatial size of the phantoms were inspired by a study of a multi-spectral dataset [27]. Figure 3 presents a visualisation of the two phantoms, each showing a distinct energy level (45keV and 70keV), highlighting the differentiation in the appearance of the phantoms and their respective objects when observed in different energy channels. Water, olive oil, nitromethane, and acetone are selected for the first phantom (Figure 3a), and methanol, ethylenediamine, aluminium, and nitrobenzene are chosen for the second phantom (Figure 3b).

To generate simulated test data, we used the 2D spectral CT phantoms and generated sinograms of 1D projections ($y_{E_{1}},y_{E_{2}},\cdots ,y_{E_{N}}$) using the geometry described below over the full angular range of $360^{\circ }$ with $1^{\circ }$ increments and corrupted these with Poisson noise using the source spectrum ($I_{0}\left(E\right)$) discussed above. If $y^{p}\left(E\right)$ is the simulated clean X-ray attenuation value for one pixel in the projections, then the noisy pixel (${\tilde{y}}^{p}\left(E\right)$) for that energy is distributed as follows:

(8) $$I_{0}\left(E\right)e^{-{\tilde{y}}^{p}\left(E\right)}∼\mathrm{Poisson}\left(I_{0}\left(E\right)e^{-y^{p}\left(E\right)}\right),$$

All noisy projections were split into 4 sets (K=4), and each of them was reconstructed with the FBP algorithm.

A linear array detector was simulated with 0.8 mm wide pixels in a 256-pixel array. The scanning geometry used a 57.50 cm distance between the X-ray source and the object and a 58.05 cm distance between the object and the detector, simulating the setup reported in [27].

### 3.2. Spectral Biological Data Acquisition

We used the data provided in [28]. The details of the acquisition procedure for the biological sample can be found in [29]. In short, the head of a lizard, approximately 17 mm in length and 10 mm in width, which provides contrast-rich views of soft tissues stained with 1% elemental iodine (I), was scanned using a HEXITEC detector [30] at a beam voltage of 50 kV. The choice of iodine is of particular interest in the preparation of the biological sample because of the presence of absorption edges located at 33.17 keV. This edge represents the K-shell binding energy of the atom, known as the K-edge, and is unique for each element [22]. The acquisition of the biological sample involved recording projection images at $2^{\circ }$ intervals throughout a $360^{\circ }$ rotation, with each projection exposed for 120 s, culminating in a total scanning time of 8 h. The energy range of the biological sample used in our experiments is between 17.5 and 43.9 keV over 96 energy bins. These 180 projections (180×80×80) were split into two sets and reconstructed using the Feldkamp–Davis–Kress (FDK; the 3D form of filtered backprojection for cone-beam imaging) algorithm [31] separately for each energy channel. Due to the limited number of projections, they were divided into 2 sets (K=2) to minimise artefacts resulting from undersampling.

To estimate the system geometry parameters needed for the reconstruction, a geometric magnification of 1.81 was established to ensure the complete and accurate projection of the biological sample onto the detection area. The configuration included a distance of 332.0 mm from the X-ray source to the centre of the object and 270.0 mm from the centre of the object to the detector. The detector itself featured a pixel size of 0.250 mm and an 80×80-pixel array.

### 3.3. Comparative Reconstruction Approaches

For comparison with our method, we also employed a traditional iterative reconstruction method that imposes a total variation [32] constraint, as well as the Low2High approach [12], both of which use all noisy projections as inputs (360 projections for synthetic datasets and 180 projections for biological samples). The total variation approach minimises the following cost function:

(9) $$x_{\mathrm{reco}}=\arg\min_{x}\left\{\frac{1}{2}{∥Ax-\tilde{y}∥}_{2}^{2}+\alpha \mathrm{TV}\left(x\right)\right\},$$

where the α parameter controls the regularisation strength and is chosen empirically for each phantom to optimise denoising performance, which requires knowledge of the clean image, which is not available in real applications.

In determining the regularisation parameters, for each spectral CT synthetic phantom, the average PSNR values of images obtained using 5 different parameters were calculated. In this process, the parameter that provided the highest average PSNR value was considered to achieve the best performance and was selected for use in the traditional reconstruction method. The determination of parameters for the biological sample was conducted through a different strategy due to the absence of ground truth. In this process, various parameters were tested, and the quality of the images in their spatial dimensions was assessed visually (although the impacts of only 3 regularisation parameters are highlighted in this paper). The parameter that provided the best image quality was selected based on this visual evaluation. This approach was applied to maximize image quality and achieve the best results in the imaging process using iterative algorithms. All experiments were performed using the Core Imaging Library (CIL) [33].

### 3.4. Network Implementation and Training

We utilised the U-Net architecture reported in [14] using PyTorch v.2.1, ref. [34] which remains state-of-the-art in many biomedical image denoising applications. U-Net was selected for its simplicity and capacity to manage the complexity of 4D data, including the spectral dimension, making it an appropriate choice for this study, particularly given the unsupervised nature of the task and the absence of both labelled and clean data in spectral imaging. We implemented both 2D and 3D U-Net architectures to accommodate the diverse data requirements. We employed a 3D convolutional neural network for biological samples and a 2D convolutional neural network for synthetic data, which requires less computational cost compared with the 3D model. Both models consist of three layers and use ReLU activation functions. The differences between these models are the dimensions of the maximum pooling and batch normalization layers. The 2D model uses MaxPool2d and BatchNorm2d, which are specialized for 2D images. In contrast, the model developed to work with 3D data includes layers such as MaxPool3d and BatchNorm3d that are specifically designed to process 3D data [35].

In the synthetic data experiment, we cropped our original images before starting the training process because having dimensions that are powers of two significantly simplifies various computational processes, especially in deep learning architectures that involve sub-sampling. Each image has 128 energy channels and 96 × 96 pixels in the spatial domain. Specifically, this training process was conducted using a block-based approach. Inputs and targets were divided into blocks with dimensions of 4×16×16, where 4 is the energy dimension. Selected blocks had a 75% overlap in both the energy and spatial dimensions.

In the experiment conducted with the biological sample, each image had 96 energy channels and 80 × 80 × 80 pixels in the spatial domain. Inputs and targets were divided into blocks with dimensions 4×16×16×16, where 4 is the energy dimension. Overlap between blocks was defined as 75% in the energy dimension (chosen to prioritize energy information) and 50% in the spatial dimension. Due to RAM constraints faced by our system, the overlap ratio in spatial dimensions was reduced relative to previous settings.

We trained both the 2D and 3D models using the Adam optimiser [36] with a learning rate of $10^{-4}$; the 2-D model was trained over 100 epochs, and the 3D model was trained over 50 epochs, both utilizing a batch size of 32.

### 3.5. Image Quality Assessment

The quality of the denoised images was assessed against the ground-truth phantoms using the SSIM and PSNR metrics applied channel-wise to the entire image. We further analysed the overall image quality by computing the mean and standard deviation of the SSIM and PSNR metrics in the energy direction. This approach allowed us to quantitatively evaluate the impact of the performance of our method across energy channels. Additionally, by examining the LACs of selected regions of interest (ROIs) of various materials across the energy channel, the accuracy of recovery of the linear attenuation coefficient profiles, which can be used to identify different materials, was assessed. To assess the noise in the selected ROIs in detail, the standard deviation of the ROIs was calculated and averaged over the energy channel. This helped to obtain an overall noise profile by quantitatively evaluating the noise variations between energy levels. To further assess the X-ray attenuation profiles for each material in the synthetic phantom, the MSE between the denoised and ground-truth profiles was computed, with both the mean and standard deviation calculated over the energy channels. Smaller values indicate better alignment with the ground truth.

Due to the lack of ground truth in the real data, we could not use the PSNR and SSIM metrics to evaluate the performance of our method on the biological sample. However, the evaluation of our approach was not only based on qualitative methods, which include attenuation profile comparison, but also incorporated quantitative measurements, including channel-wise CNR calculation. First, in the assessment, the average signals across energy channels were calculated for each specified ROI. Then, the data in these channels were flattened to obtain the standard deviation values separately, and these deviations were averaged over the energy channel to provide a representative measure of noise for the entire spectral range. The channel-wise CNR was calculated using the means and standard deviations according to Equation (7). Bold formatting in the tables is used to consistently highlight the best performance in image quality assessment, making it easy to identify the top results in all tables.

## 4. Results

We applied our new Noise2Inverse-based training method to the two synthetic phantoms and a biological sample containing a chemical tracer (iodine) and compared our approach to the Low2High method, as well as to a traditional iterative reconstruction method. The TV constraint inverse problem was solved using the FISTA [32] solver with the optimal parameters for the TV minimisation found to be 1 for the first synthetic phantom and 0.5 for the second synthetic phantom. This parameter was set at 0.035 for the biological sample. The iterative method was run for 100 iterations for phantoms and the biological sample. In the remainder of this section, the iterative method, Iterative Reconstruction with Total Variation, is referred to as IR-TV.

**Figure 4:**
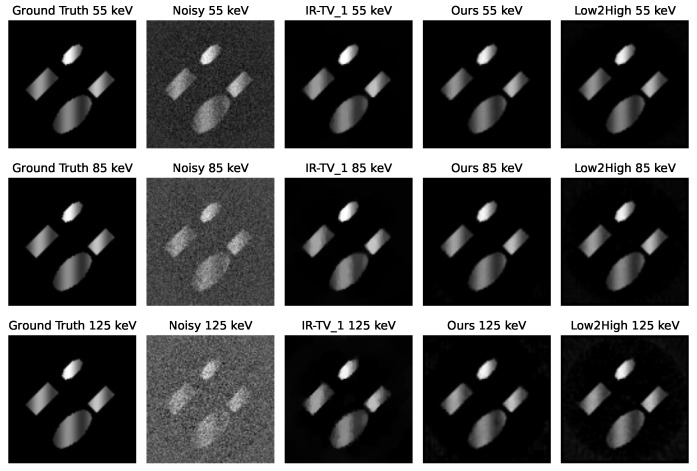
Channel-wise reconstruction for the first phantom. The first column is the ground truth, and the second column is the noisy reconstruction with FBP. The third column represents the iterative reconstruction method, and 1 indicates the alpha value selected for TV minimisation. The fourth column shows our method, and the last column shows the unsupervised Low2High method.

**Figure 5:**
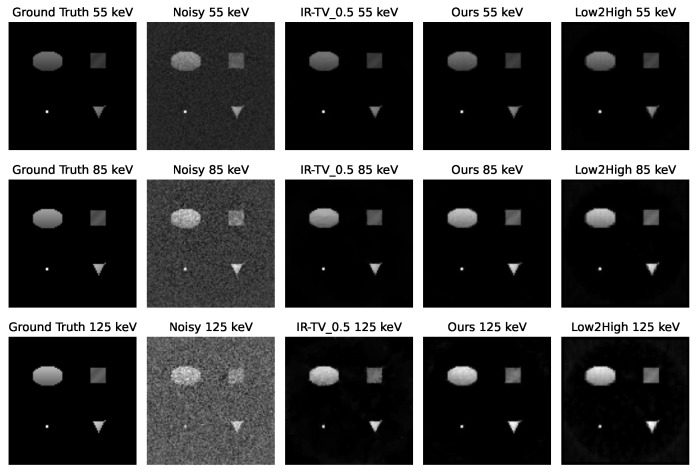
Channel-wise reconstruction for the second phantom. The first column is the ground truth, and the second column is the noisy reconstruction with FBP. The third column represents the iterative reconstruction method, and 0.5 indicates the alpha value selected for TV minimisation. The fourth column shows our method, and the last column shows the unsupervised Low2High method.

**Figure 6:**
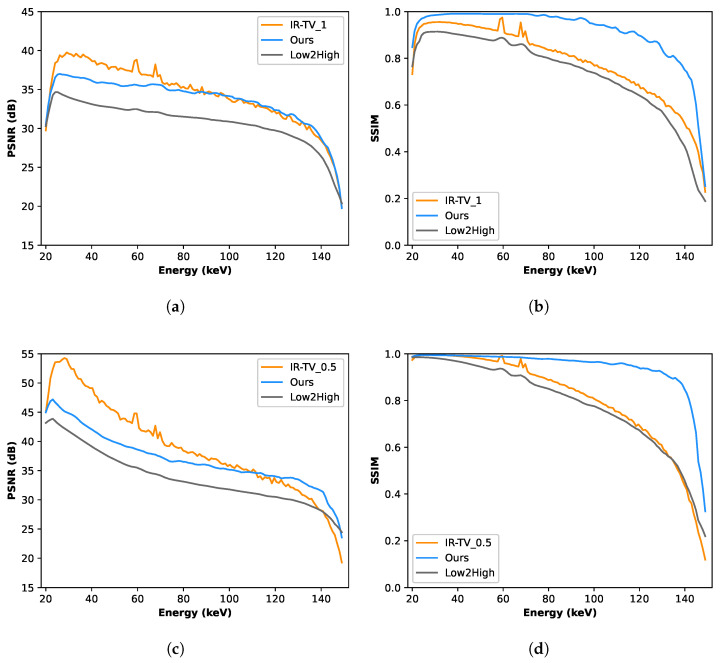
Comparative analysis using PSNR and SSIM metrics of synthetic data. These metrics are evaluated across the entire image. (**a**) Channel-wise PSNR for the first phantom; (**b**) channel-wise SSIM for the first phantom; (**c**) channel-wise PSNR for the second phantom; (**d**) channel-wise SSIM for the second phantom.

Figure 4 and Figure 5 show the denoised images of each phantom at three different energies (55, 85, and 125 keV) for all different methods. Interestingly, according to the channel-wise SSIM and PSNR metrics shown in Figure 6, our method achieved better performance, especially for high-noise (i.e., low photon count) energy channels (the low and high energy channels, where the source spectrum has limited flux), although the average PSNR performance was still found to be better for the iterative method.

The results (Table 1 and Table 2) indicate that our method exceeds the performance of the Low2High and traditional iterative methods for each phantom, as measured in average SSIM values across the energy channel. In addition, our method demonstrated comparable performance in terms of average PSNR for the first phantom; however, for the second phantom, the average PSNR was inferior to that achieved by the traditional iterative reconstruction method. Furthermore, the high standard deviation of 7.9 in the PSNR values for the IR-TV method on the second phantom indicates significant variability across the energy axis, which may contribute to the less consistent performance compared to data-driven approaches.

**Figure 7:**
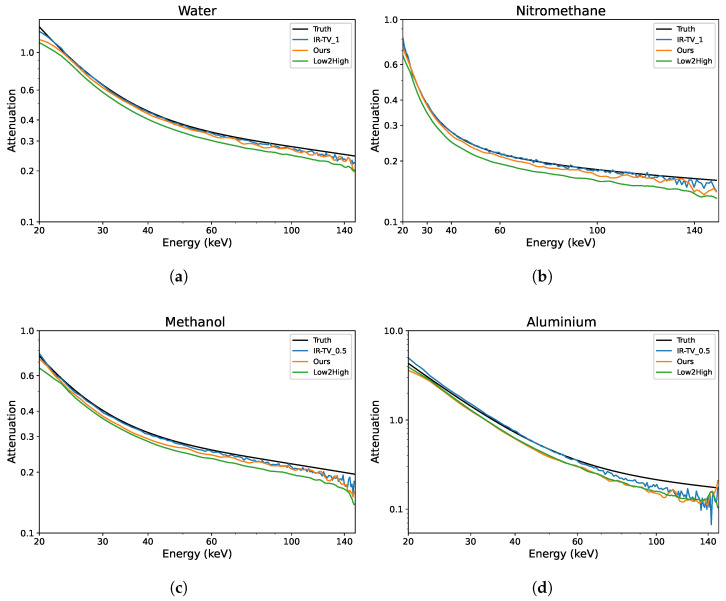
Examples of the linear attenuation coefficient of different materials over the energy channels for the synthetic data. Both axes are shown on a logarithmic scale, with tick marks manually adjusted for clarity. (**a**) Water; (**b**) nitromethane; (**c**) methanol; (**d**) aluminium.

To evaluate the denoising performance across the energy channels, ROIs were randomly selected within the objects of interest, and analysis was performed on four ROIs—two from each phantom. Each ROI was compared with the ground truth; thus, the attenuation profiles of four different materials were evaluated as illustrated in Figure 7. The LAC values were better preserved over the energy channels with the unsupervised methods (Low2High and our approach) compared to the IR-TV method in terms of noise. The LAC profile of the Low2High method is slightly lower than that of our method compared with the ground truth; this shrinking and smoothness comes from the use of the filter in the reconstruction of the Low2High method. Notably, our method exhibits a closer alignment with the ground truth over the energy channels. Despite the presence of noise within the LAC profile, the IR-TV method for each phantom yielded superior average PSNR outcomes. This is because an area was analysed across the energy channels for the LAC profile, but PSNR was averaged over the energy channels. Most importantly, with respect to the standard deviation of the pixel values (considered noise) in the selected ROIs (as seen in Table 3), unsupervised methods show lower values than the IR-TV method. Lower standard deviation values indicate reduced noise, suggesting that unsupervised methods are more effective at noise reduction, resulting in clearer and cleaner images across energy channels. The impact of the filter employed in the Low2High method is also evident in these results. Additionally, the IR-TV method exhibits higher noise levels due to the lack of regularization along the energy dimension. While over-regularization could help reduce noise, it makes the process time-consuming and less efficient. For a more detailed evaluation of the methods’ performance, we computed the MSE between the denoised attenuation profiles and the ground-truth attenuation profiles for the materials, which are shown in Figure 7. We then calculated both the mean and standard deviation of the MSE across the energy channels, and these results are presented in Table 4. While the Low2High method appears visually smoother and has a lower standard deviation, it comes with trade-offs, as both the averaged MSE and the standard deviation of the MSE indicate a greater deviation from the ground truth. This suggests that it may not be ideal for more detailed tasks, such as material decomposition, where accuracy and fine detail are critical. In contrast, our method achieves a balance between alignment with the ground truth, as indicated by reasonable MSE values, and effective noise reduction, as reflected by a moderate standard deviation. This balance suggests its ability to reduce noise while preserving key material features, making it potentially more suitable for tasks requiring precise material identification.

**Figure 8:**
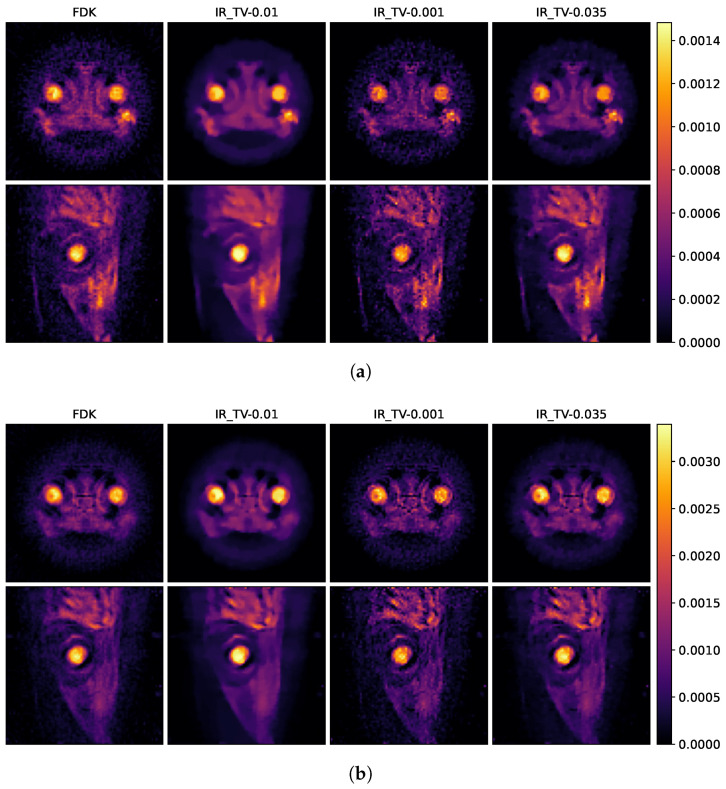
Reconstruction result of iterative methods at energies of (**a**) 25 keV and (**b**) 35 keV. The first column represents the reconstruction with full projection using FDK. The other three columns show the iterative reconstruction method, the numbers in the headings indicate the alpha values selected for TV minimisation.

**Figure 9:**
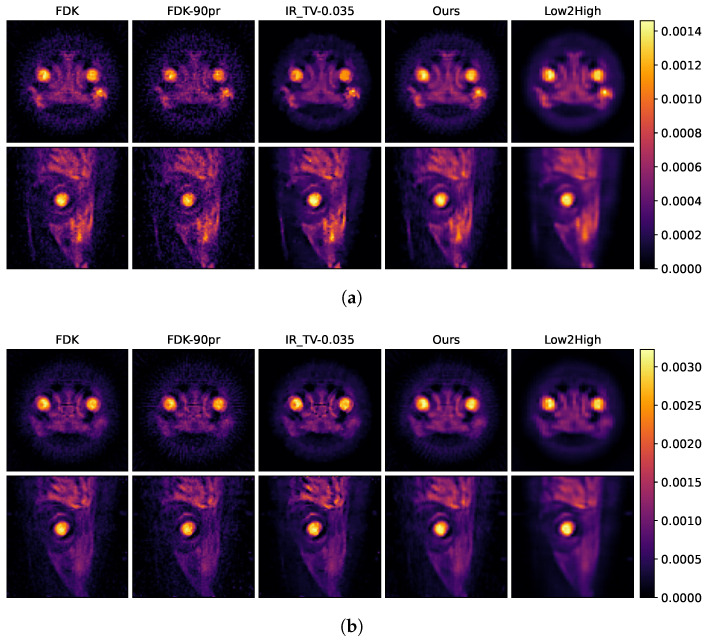
Reconstruction result of of all methods at energies of (**a**) 25 keV and (**b**) 35 keV. The first column represents the reconstruction with full projection, and the second column represents the reconstruction with half projection using FDK. The third column represents the iterative reconstruction method, and 0.035 indicates the alpha value selected for TV minimisation. The fourth column shows our method, and the fifth column shows the unsupervised Low2High method.

**Figure 10:**
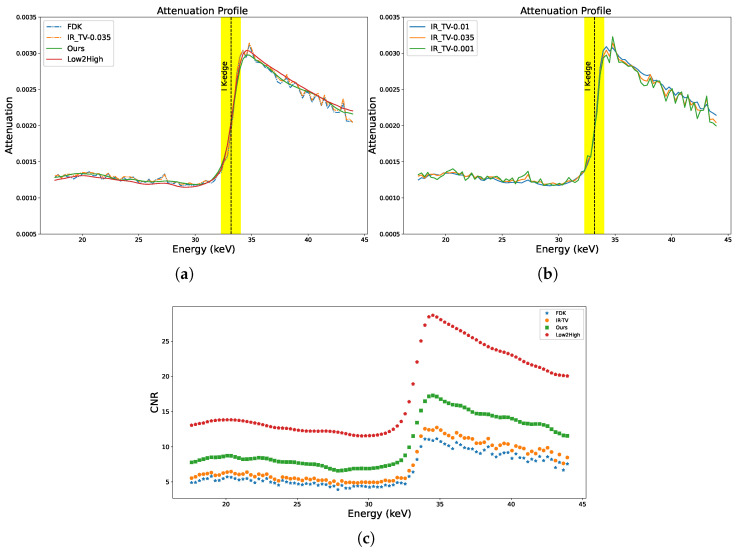
Attenuation profile across energy levels for the selected area from the lens in the biological sample. The coloured line signifies the K-edge of iodine at 33.169 keV. (**a**) All methods; (**b**) iterative methods; (**c**) calculations of CNR across channels for the iodine-stained jaw of the biological sample.

The reconstruction results of the biological sample are shown in Figure 8 and Figure 9, respectively. Figure 8 demonstrates the smoothing effects in both the spatial domains for the biological sample, depending on the selected parameter in the iterative reconstruction method. As we discussed before, various regularization parameters (0.01, 0.035 and 0.001) are determined to examine the effect of smoothness in the spatial and spectral dimensions. The selection of the regularization parameter critically influences the balance between spatial and energy dimensions. A larger regularization parameter tends to produce smoother, less detailed images in the spatial dimension but results in less noise within the energy dimension. Conversely, a smaller regularization parameter enhances sharpness and detail in the spatial dimension at the cost of increased noise in the energy dimension. Therefore, to optimise the trade-off between quality in spatial and energy dimensions, 0.035 is used for the final reconstruction for IR-TV to compare with the other methods. Figure 10b illustrates the effect of regularization parameters on the energy dimension.

In Figure 8 and Figure 9, the first row shows axial reconstructed slices, and the second row shows sagittal reconstructed slices, each at different energy levels (25 keV and 35 keV). These two energy levels (25 and 35 keV) were deliberately chosen to study the spatial effects of the iodine K-edge in the absorption spectrum. Through examination of images obtained at energy levels of 25 and 35 keV, a significant increase in the amount of attenuation after the K-edge was observed. This increase is indicative of the highly attenuated iodine marker (tracer) used in the sample.

The second column in Figure 9a,b shows examples of input used during the training process, indicating that the quality of these examples is insufficient. This becomes more evident when compared with the results of our model presented in the fourth column. The results in the fourth column visually demonstrate how our model is able to produce effective results from this initial dataset and its capacity to improve its overall performance. This comparison clearly highlights the robust nature of our model and its improvements. In the data-driven approach called Low2High investigated in this paper, the impact of the blocks used in the training process is evident; this method exhibits the poorest performance in the spatial dimension. However, as illustrated in the third column, the IR-TV method achieves significantly sharper images than the data-driven approaches in the spatial dimension.

**Figure 11:**
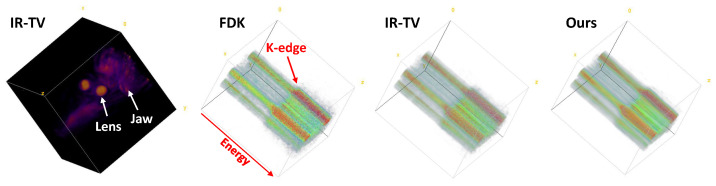
Three-dimensional visualisation of the biological sample. The image on the left depicts a 3D spatial visualisation of the biological sample reconstructed with IR-TV for an energy channel. The remaining images show a spatial 2D image slice, with the third dimension showing the energy channels for the reconstructions obtained by FDK, IR-TV, and our approach, respectively. The K-edge in the energy spectrum is shown with a red arrow, ROI’s are indicated by a white arrows. The y axis in the latter images represents the energy dimension.

Figure 10a,b show the absorption spectra of the ROIs selected from the lens of the sample for all methods (ROIs are indicated by white arrow in Figure 11). In the figure, the K-edge of iodine is clearly highlighted at 33.169 keV in the absorption spectra. Absorption edges (K-edges) serve as critical markers in chemical identification processes. Accurate and complete detection of these edges is crucial to the identification of materials.

As we can see in Figure 10a, the IR-TV method exhibits noise levels nearly comparable to those of the FDK method. Noticeably, Low2High demonstrates a much smoother profile than our method, but our method shows closer alignments. This smoothness in the attenuation profile of Low2High is due to the effect of the filter used in the reconstruction. This pattern is similarly observed in synthetic data experiments, indicating the general applicability of the method to other data types. However, since we lack a ground truth, it remains uncertain whether the attenuation profile achieved with Low2High is unbiased. This is because it was demonstrated to be biased relative to the ground truth in the synthetic data experiment. Figure 10c shows the result of the channel-wise CNR calculation for the ROI in the jaw of the biological sample (ROI is indicated by a white arrow in Figure 11). The K-edge effect is still clearly observed in the channel-wise CNR, sharply improving the image contrast. The results presented in Figure 10a,c are validated by the strong alignment between channel-wise CNR and the attenuation profile of the sample for all methods. A comprehensive assessment of image quality was performed using the average CNR calculated over energy channels. Data-driven approaches generally exhibited superior performance compared to traditional techniques (as seen in Table 5).

In order to provide a comprehensive understanding of the biological sample, Figure 11 presents a detailed 3D visualisation. The image in the first column of the figure shows a 3D visualisation of the spatial dimension of the sample, allowing for an in-depth examination of its structure. The lens and jaw of the biological sample contain K-edge material (iodine), which is clearly visible in the first column, as indicated by a white arrow. Images in the other columns, on the other hand, illustrate the energy distribution, offering insights into the sample’s energetic properties. Images in these columns are the reconstructions obtained by FDK, IR-TV, and our method, respectively. In the 3D energy mapping, a spatial slice of the sample was added to the energy profile of that dimension, and a 3D visualisation was acquired. As demonstrated by the 3D energy mapping images, our method exhibits superior performance in reducing noise in the energy dimension compared to other methods. The sudden increase in the energy spectrum of this marker is even more pronounced in the visualisation of the energy dimension. This dual visualisation approach facilitates a more thorough analysis of the biological sample’s characteristics. Considering performance in both the spatial and energy dimensions, our approach shows promising results compared to other methods.

## 5. Discussion and Conclusions

The employment of energy information in spectral CT imaging is significant, as it enables the decomposition of materials with similar attenuation properties and enhances the accuracy of material decomposition. However, the projection data are intrinsically noisy because there are not many photons within each energy channel. Here, to address the difficulty of collecting clean data, we studied the feasibility of a learning-based denoising approach that does not require additional clean and noisy training data for two synthetic phantoms and a biological sample. The image quality was preserved across spectral channels in two different spectral phantoms and one biological sample without the necessity of parameter adjustment. U-Net denoised the FBP/FDK reconstruction using a noisy sinogram splitting strategy. The self-supervised U-Net was robust to the varying noise levels of the different energy channels, especially the first and last energy channels.

Whilst the traditional total variation-constrained reconstruction was also found to perform similarly well or even slightly better than our approach in the spatial dimension, that was only achieved with significant and time-consuming parameter tuning. As the optimal parameter is highly sensitive to the image structure, this approach would not be feasible without knowledge of the ground-truth image and is, thus, not easily applicable in real applications. Furthermore, it is well known that TV regularisation leads to biased results, which can introduce further errors in the estimated spectra, which, for quantitative applications, can lead to unacceptable errors.

In this study, we have demonstrated the application of Noise2Inverse to spectral computed tomography using a block-based training approach with U-Net, showing that the use of Noise2Inverse in spectral CT imaging does offer a significant improvement in image quality. This was achieved without needing to fine tune, regularisation parameters which is a drawback of traditional iterative approaches. Some questions remain unanswered; due to the limited number of projections in the real-world experimental dataset, the number of splits was set to K=2, and this splitting strategy yielded better results than iterative approaches. However, increasing the number of splits will increase the number of training data we employ for denoising, which we believe will improve the performance of our method, as demonstrated in [13]. Unfortunately, due to the limited number of projections, this issue could not be addressed in this paper. Future studies will focus on evaluating the performance of learned spectral noise reduction techniques across various data acquisition strategies. In this context, the effects of variables such as acquisition time, the number of projections, and exposure time, on spectral data quality will be examined. Crucially, the effectiveness of energy-dimension regularization will be investigated using iterative methods and compared with learned methods. With these future studies, we aim to provide information that will contribute to the potential of spectral CT technology.

## Figures and Tables

**Table 1:** Channel-wise averaged SSIM and PSNR of different methods for the first phantom (mean ± SD).

Method	SSIM	PSNR (dB)
IR-TV_1	0.79±0.16	**34.4** ± **3.9**
Ours	**0.92** ± **0.12**	33.7±3.0
Low2High	0.74±0.18	30.8±2.7

**Table 2:** Channel-wise averaged SSIM and PSNR of different methods for the second phantom (mean ± SD).

Method	SSIM	PSNR (dB)
IR-TV_0.5	0.81±0.21	**39.0** ± **7.9**
Ours	**0.94** ± **0.10**	36.9±4.6
Low2High	0.79±0.18	33.8±4.5

**Table 3:** Channel-wise averaged standard deviation for selected ROIs of materials.

Method	Water	Nitromethane	Methanol	Aluminium
IR-TV	0.068	0.048	0.032	0.508
Ours	**0.058**	0.044	0.03	0.4
Low2High	**0.058**	**0.040**	**0.027**	**0.398**

**Table 4:** Mean and Standard Deviation of MSE over the energy channels between attenuation profiles of denoised and ground-truth images for different materials.

Method	Metric	Water	Nitromethane	Methanol	Aluminium
IR-TV	Mean	**0.00016**	**0.00003**	**0.00013**	0.00893
	SD	**0.00063**	**0.00009**	**0.00020**	0.04477
Ours	Mean	0.00080	0.00012	0.00034	0.01280
	SD	0.00458	0.00027	0.00034	0.04806
Low2High	Mean	0.00253	0.00082	0.00098	**0.00701**
	SD	0.00720	0.00145	0.00092	**0.01490**

**Table 5:** Contrast-to-noise ratio averaged over energy channels.

Method	CNR
FDK	6.62
IR-TV_0.035	7.55
Ours	10.51
Low2High	**17.41**

## Data Availability

The data used in this study comprise a hyperspectral X-ray CT biological sample dataset, which is available in [28].

## Abbreviations

The following abbreviations are used in this manuscript:

CT	Computed Tomography
PCD	Photon-Counting Detector
TV	Total Variation
PSNR	Peak Signal-to-Noise Ratio
MSE	Mean Square Error
SSIM	Structural Similarity Index
CNR	Contrast-to-Noise Ratio
SD	Standard Deviation
LAC	Linear Attenuation Coefficient
IR	Iterative Reconstruction
